# How Does the Parent–Adolescent Relationship Affect Adolescent Internet Addiction? Parents’ Distinctive Influences

**DOI:** 10.3389/fpsyg.2022.886168

**Published:** 2022-06-07

**Authors:** Huaiyuan Qi, Qinhong Kang, Cuihua Bi

**Affiliations:** School of Psychology, Sichuan Normal University, Chengdu, China

**Keywords:** adolescents, parent–adolescent relationship, internet addiction, perceived social support, dual system of self-control

## Abstract

Although previous research has demonstrated that parent–adolescent relationships have a significant effect on adolescent Internet Addiction (IA), the mechanisms underlying these associations and parental differences in these effects have received insufficient attention. We investigated the mediating role of Perceived Social Support and Dual System of Self-Control (DSSC) in the relationship between Father-Adolescent Relationships/Mother-Adolescent Relationships (FAR/MAR) and adolescent IA, as well as the differences in the effects of FAR and MAR. A cross-sectional survey of 732 Chinese adolescents was conducted using the Adolescent Pathological Internet Use Scale, Parent–Adolescent Relationship Scale, Multidimensional Scale of Perceived Social Support, and Dual System of Self-Control Scale. Multiple linear regression analysis, Pearson correlation analysis and structural equation modeling were used. The results of structural modeling analysis showed that neither FAR nor MAR directly predicted adolescent IA. In contrast, FAR/MAR had an impact on adolescent IA mainly through the mediating effects of Perceived Social Support and Impulsive System. Furthermore, in the relationship between FAR/MAR and adolescent IA, the Impulsive System and Perceived Social Support both served as chain mediators, as did Perceived Social Support and the Reflective System. And more importantly, unlike FAR, MAR affects adolescent IA through the mediating effect of the Reflective System. Multiple linear regression showed that the regression coefficient of MAR on adolescent IA had stronger significance compared to FAR, MAR is deserving of more attention than FAR. These findings contribute to our understanding of the mechanisms underlying the association between FAR/MAR and adolescent IA and suggest that family relationship-focused training approaches are critical for suppressing adolescent IA. These interventions should be tailored to the unique circumstances of each family.

## Introduction

Internet devices (e.g., computers, smartphones, and tablets) have become increasingly popular among adolescents in recent years, and an increasing number of adolescents are overusing Internet devices, which has resulted in Internet addiction (IA) becoming a widespread problem worldwide (Talis, 2022). IA has been defined differently by various researchers. In Goldberg (1996), coined the term “Internet Addiction Disorder (IAD)” to describe the effect of excessive Internet use on people’s daily lives in the absence of addictive substances. Then some researchers define IA as a behavioral disorder that is unrelated to addictive substances and is therefore a typical mental illness disorder (Young, 2004). Davis (2001) proposed a cognitive-behavioral model, defined IA as problematic Internet use (PIU), classified PIU into general and specific pathological Internet use, and developed the problematic Internet use scale (PIUS) to assess individuals’ irrational perceptions and usage behaviors. On the other hand, Young (1998) developed a questionnaire to assess IA behaviors based on the DSM-IV diagnostic criteria for pathological gambling; additionally, Li and Yang (2007) developed the Adolescent Pathological Internet Use Scale (APIUS) based on Davis’ PIU model. Although there is no universally accepted definition of IA, existing definitions and questionnaires place a premium on describing people’s emotional, attitude, and behavioral dependence on the Internet, as well as the negative consequences of this dependence on their lives.

Numerous studies have established that adolescents who suffer from IA have poorer mental health than adolescents who do not suffer from IA (Lauricella et al., 2014). IA demonstrated significant negative correlations with adolescents’ self-esteem (Fioravanti et al., 2012; Peng et al., 2019), psychological well-being (Jia et al., 2017), and peer aggression (Jia et al., 2018). And IA has been shown to impair academic performance, anxiety disorders, feelings of stress, depression, and aggression in adolescents (Ha and Hwang, 2014; Shek and Yu, 2016; Cerniglia et al., 2017; Wang C. Y. et al., 2017; Zhao et al., 2022), thereby inhibiting adolescents’ positive psychological development. More importantly, the prevalence of IA has remained high in adolescents over the last decade. The prevalence of IA among adolescents varies considerably across the globe, ranging from 26.8 percent in Hong Kong (Chung T. W. et al., 2019) to 14.7 percent in Taiwan (Lin et al., 2018), 1.5–8.2 percent in Europe and the United States (Kuss et al., 2014), 6.5 percent in Switzerland (World Health Organization, 2015), and 40.64 percent in mainland China (Bu et al., 2021). Thus far, the global situation for adolescents with IA has deteriorated, with prevalence rates ranging from 12.6 to 67.5% (Kuss et al., 2021). Why does the prevalence of IA vary by country or region among adolescents? Some researchers conducted a meta-analysis of the prevalence of IA in 31 countries or regions and discovered a significant correlation between the prevalence of IA and lower life satisfaction, increased air pollution, increased transportation commuting time, and lower national income (Cheng and Li, 2014). Cheng and Li also argue that as a country or region advances technologically, it may result in a sustained increase in the prevalence of IA. On the other hand, Blachnio et al. (2019) argue that the cultural presence of denial of one’s addiction and loss of control over one’s online time are significant manifestations of the IA epidemic (e.g., Taiwan).

Gaming content, social content, short video content (e.g., Tik Tok), and news and information on Internet devices are all intrinsically appealing and can provide instant feedback to users of all ages, making them happy and enjoyable (Peris et al., 2020). While any group has the potential to develop an addiction to the Internet, there are several reasons why adolescents are more likely to develop an addiction than other age groups: (1) More eager for attention: Compared to other age groups, adolescents are more eager for attention from others to satisfy their sense of belonging and self-expression (Kim and Kim, 2015), and, in comparison to real life, the Internet provides a platform for adolescents to demonstrate themselves, and they are more willing to establish and sustain social relationships on online platforms, making them more likely to generate IA (Seidman, 2013). (2) More impulsive: there is evidence that impulsivity increases from childhood to adolescence and then declines (Rosenbaum and Hartley, 2019), and that increased impulsivity in adolescents may be related to ventral striatum hyperresponsivity (Sherman et al., 2018). Comparing groups revealed that IA individuals were more impulsive than healthy controls (Li et al., 2021), and behavioral experiments also indicate that IA individuals are more focused on immediate gratification (Wang L. et al., 2017). These findings suggest that impulsivity is strongly associated with IA in adolescents. (3) More negative coping styles: Adolescents undergo rapid physical, emotional, intellectual, and life development and are susceptible to a variety of negative events such as peer pressure, academic stress, school bullying, emotional difficulties, and social discrimination (Kuss et al., 2013; Lin et al., 2018; Chung S. et al., 2019). Adolescents, on the other hand, often struggle to actively deal with these life frustrations, and instead turn to the Internet environment to vent and escape from real-world problems, eventually developing an unhealthy reliance on the Internet. As a result, adolescents have a higher risk of developing IA than other age groups (Anderson et al., 2017).

### Parent–Adolescent Relationship and Adolescent Internet Addiction

Previous research has indicated that the primary causes of IA in adolescents are either individual factors (e.g., self-control, personality traits) or environmental factors (e.g., family economic environment, school environment) (Xu et al., 2012; Zhou et al., 2017). However, an increasing number of researchers have discovered that relationships between parents and adolescents in the family environment may be a significant influence factors for adolescent IA. Through a questionnaire study, Park et al. (2008) discovered significant differences in parent-child relationships between three groups: IA, addictive tendency, and non-addiction. *Post hoc* comparisons revealed that the parent–child relationship was significantly less positive in the addiction and addictive tendency groups compared to the non-addiction group. Additionally, Xu et al. (2014) examined the parent–child relationships and family environment of adolescent parents. They discovered that, while family socioeconomic status had no effect on adolescent IA prevalence, the quality of the parent-adolescent relationship had a significant negative effect on adolescent IA prevalence. In conclusion, while a positive parent-child relationship can help adolescents feel accepted and act as a protective factor against IA (Ahmadi and Saghafi, 2013), prolonged parent–child conflict can cause adolescents to perceive themselves as rejected and act as a risk factor for IA (Ko et al., 2015). Thus, strengthening the parent-child relationship can help adolescents develop adaptive functioning and reduce their risk of developing IA (Kapetanovic et al., 2019; Skinner et al., 2021). For instance, Liu et al. (2015) discovered that family group therapy can address adolescents’ psychological needs by enhancing parent-adolescent communication and facilitating parent–adolescent relationships, ultimately resulting in effective treatment for adolescent IA.

Recently, researchers have begun to investigate the mediating effects of parent–adolescent relationships on adolescent IA in order to better understand how parent–adolescent relationships affect IA. There is evidence that factors such as emotion regulation ability (Wang J. et al., 2018) and peer relationships (Ding et al., 2017) mediate the relationship between parent-adolescent relationships and adolescent IA. The Problem-Behavior Theory (PBT) aims to explain how parent-adolescent relationship further influences adolescent IA as a risk behavior by altering individual characteristics of adolescents (Jessor, 2014). PBT recognizes that adolescents’ problem behaviors (e.g., alcohol abuse, violence, and IA) are the result of the relationship of environmental and individual factors. Family is the primary environment in which adolescents live, and parenting, supervision, and behavioral control interact with adolescents’ traits, thinking, and emotions to influence the quality of parent-adolescent relationships (Mo et al., 2018; Chung T. W. et al., 2019; Martins et al., 2020). The quality of the parent–adolescent relationship may enhance adolescents’ perceptions of social support and self-control, thereby influencing their IA (Li et al., 2017; Shek et al., 2018). With the increasing prevalence of adolescent IA in recent years, the relationship between the parent-adolescent relationship and adolescent IA has also been explored to some extent. However, these previous studies made little distinction between father–adolescent relationships (FAR) and mother–adolescent relationships (MAR), and thus do not know whether FAR/MAR both influence adolescent IA in the same way. Previous empirical studies on FAR/MAR and adolescent IA have found that, while increased father control over adolescent behavior predicted a slower decline in adolescent IA, increased maternal psychological control predicted a faster decline in IA (Shek et al., 2018). Additionally, Xu et al. (2014) concluded that relationships between mothers and adolescents are more likely to influence adolescent IA. Therefore, the first aim of this study was to distinguish FAR from MAR to explore how the direct and mediating effects of FAR and MAR differ in influencing adolescent IA.

### The Mediating Role of Perceived Social Support

Perceived Social Support is a subjective emotional experience, and the more support and understanding an individual receives from family, peers, teachers, and others, the stronger the subjective social support (Kang et al., 2018). Adolescents generally receive social support from members of their environment, such as family, school, and community, and thus their Perceived Social Support is classified into three categories: family support, peer support, and other support (Hyun et al., 2015; Wang Y. et al., 2017). Adolescence is a period of rapid physical and psychological development during which adolescents frequently exhibit defiance, emotional instability, and difficulties with adaptation. Relational Regulation Theory (RRT) suggests that Perceived Social Support is always associated with good and healthy emotions and behaviors. Perceived Social Support is a positive experience resulting from ordinary and emotionally consequential conversations and shared activities that can effectively regulate adolescents’ feelings, thoughts, and actions, buffer adolescents from stress, promote mental health, and avoid problematic behaviors (Lakey and Orehek, 2011). Numerous studies have discovered that adolescents with low Perceived Social Support have impaired emotional regulation and report feelings of loneliness (Wang J. et al., 2018), depression (Ren et al., 2018; Wang P. et al., 2018), and psychological distress (Ren et al., 2018; Zhang et al., 2018). Although no empirical study has examined the relationship between Perceived Social Support, FAR/MAR, and adolescent IA directly, some indirect evidence suggests that Perceived Social Support mediates these associations. On the one hand, FAR/MAR is the primary channel through which adolescents access Perceived Social Support (Shaheen et al., 2019), and both daily positive parent-adolescent communication and behavioral relationships help adolescents increase their Perceived Social Support (Taylor et al., 2015). On the other hand, existing studies have found that Perceived Social Support can significantly and negatively predict adolescent IA (Karaer and Akdemir, 2019). In a family setting, elevated Perceived Social Support can be effective in preventing IA in adolescents (Gunuc and Dogan, 2013). Thus, FAR/MAR is associated with Perceived Social Support, which in turn may be associated with adolescent IA.

### The Mediating Role of Dual System of Self-Control

Hofmann et al. (2009) proposed the Dual-System of Self-Control Model, arguing that a complete model of self-control should include both the Impulsive and Reflective Systems. The Impulsive System is a relatively fast processing method that rarely requires cognitive processing or attentional resources. It is an automatic behavioral schema that individuals gradually form based on their previous behavioral patterns and long-term learning experiences. The reflective System, which processes information in the opposite direction of the impulsive system, is primarily responsible for restraining an individual’s tendency to react impulsively and automatically through the establishment of high-level goals for assessing, monitoring, and managing behavior, and its operation requires the involvement of individual volitional effort and attentional resources (Lieberman, 2007). The system confers a greater degree of flexibility and control over decision-making and behavior, overcoming impulsive responses elicited by stimuli or temptations. Individual self-control is achieved through the manipulation of executive functions, which enable individuals to make deliberate judgments and assessments that either inhibit or overwhelm impulsive behavior (Hofmann et al., 2009; Gillebaart, 2018).

Puberty has been shown to be strongly associated with impulsivity (Niv et al., 2012). In adolescents, a decrease in the thickness of the cerebral cortex in the area of value selection is indicative of impulsivity (Pehlivanova et al., 2018). Additionally, there is an upward trend in executive functions among adolescents as they transition from adolescence to adulthood (Friedman et al., 2016). In general, the relationship of impulsivity and executive function results in significantly lower self-control in adolescence than in adulthood (Meldrum et al., 2012; Oliva et al., 2019). Numerous studies have established a link between the DSSC, Parent–Adolescent Relationship, and IA. For instance, Niu et al. (2020) discovered a positive correlation between self-control and the Parent–Adolescent Relationship and a negative correlation between self-control and adolescent problematic internet use. Additionally, self-control mitigates the effect of the Parent–Adolescent Relationship on problematic adolescent internet use. However, no study has examined the mediating role of DSSC to our knowledge. On the one hand, the relationship between a parent and an adolescent has a significant impact on adolescents’ impulsivity and executive functioning (Fay-Stammbach et al., 2014; Bennett and Blissett, 2017). Adolescents’ self-control is highly dependent on the parent–adolescent relationship (Brody et al., 2005; Liu et al., 2019). On the other hand, increased impulsivity (Babakr et al., 2019; Zhang Y. et al., 2021), as well as a deficiency in executive function (Li et al., 2014; Fumero et al., 2018; Kuo et al., 2018), are also major contributors to IA in the adolescent population. Thus, FAR/MAR is associated with DSSC in adolescents, which may be associated with IA.

Additionally, Perceived Social Support has been demonstrated to have a significant positive predictive effect on adolescent self-control. According to the Dual-System Model of Self-Control, social support may reduce impulsivity and increase executive function activation in adolescents (Sims et al., 2011; Khoo and Yang, 2020). FAR and MAR may affect DSSC via Perceived Social Support, which in turn affects adolescent IA. As a result, Perceived Social Support and DSSC may function as a chain mediating mechanism in FAR/MAR and adolescent IA.

### The Present Study

**FIGURE 1:**
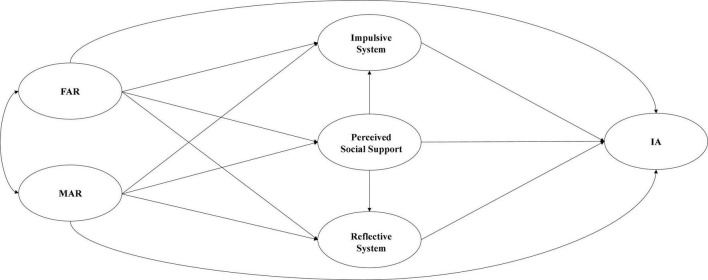
The hypothesis model.

The purpose of this study was to examine how FAR/MAR affects adolescent IA. This study investigates the mediating effects of Perceived Social Support and DSSC on FAR/MAR and adolescent IA, as well as the differences in the effects of FAR and MAR on adolescent IA. To our knowledge, this is the first empirical study to examine the roles of FAR/MAR, Perceived Social Support, and DSSC in IA concurrently. The Hypothesis Model is illustrated in Figure 1. It is based on the RRT, PBT, and Dual-System of Self-Control Model (Hofmann et al., 2009; Lakey and Orehek, 2011; Jessor, 2014). We can hypothesize that (1) FAR/MAR has a significant negative predictive effect on adolescent IA; (2) Perceived Social Support mediates the effect between FAR/MAR and adolescent IA; (3) DSSC mediates the effect between FAR/MAR and adolescent IA; (4) Perceived Social Support and DSSC act as a chain mediator between FAR/MAR and adolescent IA; (5) The effect of MAR on adolescent IA was stronger compared to FAR.

## Materials and Methods

### Participants

A total of 976 Chinese adolescents were recruited from two public schools in Sichuan Province. Before beginning the study, we obtained informed consent from the adolescents, their guardians, and teachers. Adolescents could stop filling out the questions if they felt uncomfortable with them. A total of 732 valid questionnaires were returned after excluding non-completed questionnaires. The sample included 317 boys and 415 girls, ranging in age from 11 to 16 years (*M_*age*_* = 13.83 years, *SD* = 1.20 years). Of these, 36 (4.9%) were 11-year-old students, 44 (6.0%) were 12-year-old students, 183 (25%) were 13-year-old students, 280 (38.3%) were 14-year-old students, 121 (16.5%) were 15-year-old students, and 68 (9.3%) were 16-year-old students. All students completed the questionnaire in a quiet classroom at the school.

### Sample Size Determination

Considering the structural equation modeling approach used in this study, the sample size at the time of data analysis needed to meet the criterion of matching at least 10 participants for each free parameter (Bentler and Chou, 1987; Hu and Bentler, 1999). Because the Hypothesis Model contains 58 free parameters and the Correction Model contains 55 free parameters in this study, the minimum sample size should be greater than 580 participants. This study’s sample size was adequate.

### Measurement

#### Parent–Adolescent Relationship Scale

The Parent–Adolescent Relationship Scale (PARS scale), developed by Buchanan et al. (1991), is widely used to assess parent-adolescent relationships. Parent–child relationships span a variety of dimensions, most notably attachment, parenting styles, parent–child communication, parent–child bonding, and parent–child conflict (Armstrong et al., 2018; River et al., 2022). The PARS used in this study focuses on parent–child communication and bonding and reflects the status of FAR and MAR by inquiring about adolescents’ communication and bonding with their fathers and mothers. The scale contains a total of 20 items and can be divided into two subscales: FAR (10 items, e.g., “Do you feel comfortable and natural when you express your emotions to your dad?”) and MAR (10 items, e.g., “When you want to talk to your mom, will she be willing to talk to you?”). Respondents were asked to rate the extent to which each item was true for them on a five-point scale (1 = strongly disagree, 5 = definitely applies). We summed items in each subscale, with higher scores indicating higher levels of parent–adolescent relationship. This scale has shown good reliability and validity in Chinese adolescents (Zhang et al., 2011). The Cronbach’s α coefficient in our study was 0.85 (FAR) and 0.89 (MAR).

#### Adolescent Pathological Internet Use Scale

The Adolescent Pathological Internet Use (APIU) scale developed by Li and Yang (2007) is widely used to assess IA. The scale contains a total of 38 items and can be divided into six subscales: salience (3 items, e.g., “Once I’m online, I don’t think about anything else”), tolerance (5 items, e.g., “I would rather hold back my bowel movements in order to stay online”), withdrawal symptoms (11 items, such as “When I can’t go online, I really want to know what’s happening online”), mood alteration (5 items, such as “When I’m depressed, going online can make me feel better “), social comfort (6 items, e.g., “I feel more comfortable when I communicate with others online”) and negative outcomes (8 items, e.g., “I sometimes skip class to go online “). Respondents were asked to rate the extent to which each item was true for them on a five-point scale (1 = never, 5 = always). We summed items in each subscale, with higher scores indicating higher levels of IA. This scale has shown good reliability and validity in Chinese adolescents (Liu et al., 2012). The Cronbach’s α coefficient in our study was 0.94.

#### Multidimensional Scale of Perceived Social Support

The Chinese version of the Multidimensional Scale of Perceived Social Support (MSPSS) is used to assess Perceived Social Support (Zimet et al., 1988). The scale contains a total of 12 items and can be divided into three subscales: family (four items, e.g., “My family can help me in a practical and concrete way”), friends (four items, such as “My friends can really help me”), and significant others (four items, such as “My teachers, relatives, and classmates are there for me when I have problems”). Respondents were asked to rate the extent to which each item was true for them on seven-point scale (1 = Very strongly disagree, 7 = Very strongly agree). We summed items in each subscale, with higher scores indicating higher levels of Perceived Social Support. This scale has shown good reliability and validity in Chinese adolescents (Wang L. et al., 2017). The Cronbach’s α coefficient in our study was 0.86.

#### Dual System of Self-Control Scale

The Chinese version of the Dual System of Self-Control (DSSC) scale is used to assess self-control ability, and it contains two subscales, impulse system and Reflective System (Xie et al., 2014). The impulse system subscale includes subscales: impulsive (six items, e.g., “I often do or say things without thinking”); easy distraction (three items, e.g., “I often feel unable to complete my tasks”); and delay gratification (three items, e.g., “I can’t save money for future purchases”). The Reflective System subscale includes two subscales: problem-solving (six items, e.g., “I will try everything to deal with this”) and future time view (three items, e.g., “I think we should plan our day in the morning”). Respondents were asked to rate the extent to which each item was true for them on five-point scale (1 = not at all true, 7 = very true). The higher the score on the impulse system subscale, the stronger the factors of impulsiveness, distraction, and delay gratification, and the weaker the self-control ability. The higher the score in the control system subscale, the more likely the problem is solved satisfactorily, the stronger the future time view, and the stronger the self-control. This scale has shown good reliability and validity in Chinese adolescents (Wang L. et al., 2017). The Cronbach’s α coefficient in our study was 0.82.

### Data Analysis

Statistical analysis was performed using SPSS 23.0 software and Amos 26.0 software. It was divided into the following steps: (1) Data standardization and descriptive statistics (including the mean, standard deviation, Cronbach’s α, and correlation for each variable) were performed using SPSS software. (2) Multiple regression analysis using SPSS software was used to compare the effects of FAR and MAR on adolescent IA. (3) Structural equation modeling (SEM) was conducted to test the mediating role of preceived social support and DSSC between FAR/MAR and adolescent IA (with Maximum Likelihood estimation). Where the Chi-square to degrees of freedom ratio (*x^2^/df)* < 5, the comparative fit index (CFI) and Tucker-Lewis Index (TLI) indices were above 0.90, and the standardized root mean square residual (SRMR) and the root mean square error of approximation (RMSEA) were less than 0.08 show good model fit (Bentler and Chou, 1987). (4) Bias was corrected for by a bias-corrected non-parametric percentile bootstrap method with 5000 replicate samples using 95% confidence intervals (CI). Indirect effects were significant if the 95%CI did not include zero (Preacher and Hayes, 2008).

### Normal Distribution and Multicollinearity

We used the Kolmogorov–Smirnov test to determine whether each variable was normally distributed on 732 valid samples, and the results indicated that each variable was normally distributed with a two-sided significance range of 0.058–0.18, implying that each variable was normally distributed. Additionally, the variance inflation factor (VIF) is a parameter that indicates the degree of cointegration in a multiple linear regression model, with tolerance equal to 1/VIF. If the VIF of the independent variables is greater than 5 and the tolerance value is greater than 0.2, the model is considered to have severe multicollinearity (Akinwande et al., 2015). The VIFs of the variables in this study ranged from 1.011 to 1.827; the tolerance ranged from 0.547 to 0.998, indicating that the model was not significantly multicollinear.

### Item Parceling

The FAR and MAR are both one-dimensional instruments, and the questions are highly homogeneous. To avoid measurement error inflation of latent variables, which reduces the model’s fit, the factorial algorithm method was used in this study to package the FAR and MAR questions, and the ten FAR/MAR questions were combined into two questions each (Rogers and Schmitt, 2004). After factor analysis, the questions with the highest factor loadings were included as anchor items in the package, followed by the questions with the next highest factor loadings in reverse order according to the direction of the balance, and the score for each question combination after the package was equal to the average of the questions in the package. Due to the fact that the other questionnaires have their own dimensions, there is no reason to use the factorial algorithm method.

### Control Variables

This study includes two demographic variables as control variables: age and gender (1 = male, 2 = female). The demographic control variables were chosen based on the findings of the study. To begin, adolescents may exhibit greater impulsivity than other age groups due to a more active nervous system, specifically the ventral striatum, during adolescence (Sherman et al., 2018; Rosenbaum and Hartley, 2019). Additionally, there is a non-linear increase in adolescents’ self-control throughout adolescence, which means that adolescents may have varying levels of self-control at various ages (Casey, 2015). Second, girls have greater self-control than boys among adolescents, which may be explained by the fact that girls are less impulsive during adolescence (Chapple and Johnson, 2007) and are better at contemplation and reflection (Burwell and Shirk, 2007). As a result of the preceding study, we used age and gender as control variables in our study and assigned them to the column of independent variables in multiple regression analysis. In SEM analysis, we assigned the two variables age and gender to the three latent variables: Impulsive System, Reflective System, and IA (Yang et al., 2010).

## Results

### Descriptive Statistics and Correlations

Table 1 contains the means, standard deviations, and correlation coefficients for each variable. The results indicated that Adolescent IA was significantly positively correlated with Impulsive System and negatively correlated with FAR/MAR, Perceived Social Support, and Reflective System; FAR/MAR was significantly positively correlated with Perceived Social Support, Reflective System, and Impulsive System; Perceived Social Support was significantly positively correlated with Reflective System and negatively correlated with Impulsive System; Impulsive System was significantly positively correlated with Reflective System; Impulsive System was significantly Additionally, the correlation coefficients between the main variables ranged between 0.34 and 0.55 in absolute value, and the significance coefficients between all variables were less than 0.001.

**TABLE 1:** Descriptive statistics and correlations for key variables (*N* = 732).

Variables	Age	Gender	IA	FAR	MAR	Perceived social support	Impulsive system	Reflective system
Age								
Gender	–0.01							
IA	–0.10**	–0.06						
FAR	0.07	–0.01	–0.42***					
MAR	0.10**	–0.01	–0.43***	0.55***				
Perceived social support	0.07	0.00	–0.49***	0.55***	0.51***			
Impulsive system	–0.07	0.03	0.52***	–0.42***	–0.38***	–0.47***		
Reflective system	0.05	0.01	–0.42***	0.38***	0.44***	0.49***	–0.34***	
*M*	13.83	—	2.53***	2.87***	3.10***	4.52***	2.74***	3.32***
*SD*	1.20	—	0.75***	0.99***	1.09***	1.44***	0.88***	0.79***

**p < 0.01, ***p < 0.001.

### Multiple Linear Regression Analysis

Multiple linear regression analysis was used to compare the direct effects of FAR and MAR on adolescent IA while accounting for demographic and other primary variables. Clogg et al. (1995) contended that when variables are standardized to unify their scales, the magnitude of the effects of various independent variables on the dependent variable can be compared using regression coefficients. However, this comparison is not absolute; regression coefficients represent differences in the slopes of the various variables, which means that when the coefficient for variable an is greater than the coefficient for variable b, it indicates that changes in variable a have a greater effect on the dependent variable than changes in variable b. Therefore, after standardizing all variables, we used the demographic variables and the main variables including FAR/MAR as our independent variables and adolescent IA as the dependent variable.

The model’s results were summarized in Table 2. The fit of the model was satisfactory (*F* = 68.016, *p* < 0.001). The standardized *R*^2^ value was 0.397, indicating that the independent variables could account for 39.7 percent of the variance in the model IA. In addition, the results show that FAR had no effect on adolescent IA (β = –0.071, *t* = –1.88, *p* = 0.061), whereas MAR had a significant effect on adolescent IA (β = –0.108, *t* = –2.904, *p* = 0.003). In comparison to FAR, the regression coefficient of MAR on adolescent IA was larger and more significant. This suggests that mothers may exert a greater inhibitory effect on adolescent IA than fathers do in this model condition, a finding that is consistent with previous research (Xu et al., 2014; Shek et al., 2018). It is worth noting that, while the regression coefficients for MAR were greater than those for FAR, this does not imply that MAR had a greater effect on adolescent IA under any condition. More precisely, it means that for each unit increase in MAR, the propensity for adolescent IA may decrease by 0.108 units, when these independent variables are taken into account. Relatively, due to the non-significant regression coefficients for FAR, there may not be a significant change in adolescent IA propensity for each unit of FAR enhancement. Which of the following has a greater impact on adolescent IA: MAR or FAR? It may produce inconsistent results under different conditions, which may be influenced by how the parent-adolescent relationship is measured (Li et al., 2014; Kim and Kim, 2015), necessitating additional research.

**TABLE 2:** Multiple linear regression analysis (*N* = 732).

Independent variables	β	*T*	95% CI		Tolerance	VIF
			Lower	Upper		
Age	–0.049	–1.683	–0.088	0.007	0.989	1.011
Gender	–0.074	–2.563*	–0.264	–0.035	0.998	1.002
FAR	–0.071	–1.880	–0.146	0.003	0.582	1.717
MAR	–0.108	–2.904**	–0.185	–0.038	0.598	1.671
Perceived social support	–0.172	–4.421***	–0.251	–0.097	0.547	1.827
Impulse system	0.317	9.370***	0.248	0.382	0.727	1.376
Reflective system	–0.147	–4.286***	–0.217	–0.082	0.704	1.421

*p < 0.05, **p < 0.01, ***p < 0.001.

### Structural Equation Model Analysis

Due to the inconsistent range of scores across questionnaires, and to avoid impairing model fit by inflating the measurement error of the variables, this study transformed all variables into standardized variables with a mean of 0 and a standard deviation of 1 before conducting SEM analysis (Wang and Wang, 2019).

**FIGURE 2:**
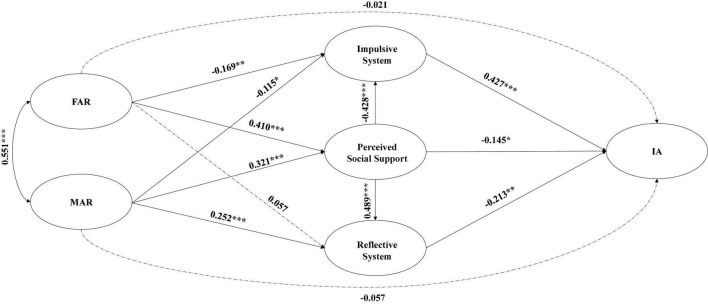
Hypothesis model. The dashed lines indicate paths with non-significant regression coefficients; control variables and precursor measures are not shown.

**FIGURE 3:**
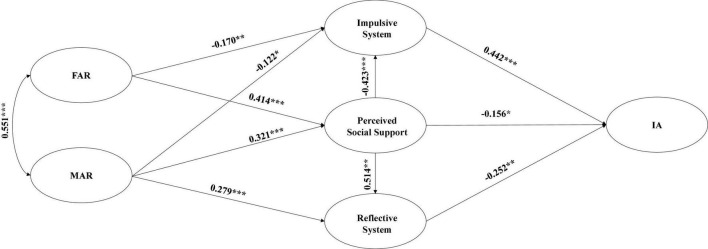
Correction model.

The test results of the Hypothesis Model are presented in Table 3 and Figure 2. where the regression coefficients of FAR on adolescent IA (β = –0.02, *p* > 0.05, 95% CI = [–0.12, 0.08]) and MAR on adolescent IA (β = –0.06, *p* > 0.05, 95% CI = [–0.15, 0.04]) were too small to be significant; the regression coefficients of FAR on Reflective System regression coefficient was small (β = 0.06, *p* > 0.05, 95% CI = [–0.05, 0.18]) with p-values greater than 0.05 and 95% CI including 0, all of which were non-significant paths. As a result, these paths have been eliminated from the Correction Model. The Correction Model’s test results are depicted in Figure 3.

**TABLE 3:** Model fitness.

Models	*x^2^/df*	*p*	△*x^2^(*△df*)*	CFI	TLI	RMSEA	SRMR
Hypothesis model	2.969	<0.001	3.712(3)	0.980	0.974	0.052	0.039
Correction model	2.935	<0.001		0.980	0.975	0.051	0.040

The difference in cardinality between the Hypothesis Model and Correction Model was *x^2^* = 3.712, *p* < 0.05. As shown in Table 3, the Correction Model fit well and all criteria were met (Bentler and Chou, 1987). SEM and bootstrap analysis (5000 replicate samples) were used to validate the hypothesized mediated paths. As shown in Table 4, (1) in terms of total effect, both FAR and MAR had a significant negative predictive effect on adolescent IA [FAR: β = –0.271, 95% CI: (–0.342) – (–0.204); MAR: β = –0.276, 95% CI: (–0.347) – (–0.204)]. (2) In terms of direct effects, neither FAR (*p* > 0.05) nor MAR (*p* > 0.05) had a significant direct effect on adolescent IA, suggesting that FAR and MAR affect adolescent IA primarily through indirect effects (Preacher and Hayes, 2008). (3) In terms of indirect effects, the mediating effect of Perceived Social Support between FAR/MAR and adolescent IA was significant [FAR: β = –0.065, 95% CI: (–0.133) – (–0.004); MAR: β = –0.050, 95% CI: (–0.105) – (–0.004)]; Impulsive System had a significant mediating effect between FAR/MAR and adolescent IA [FAR: β = –0.075, 95% CI: (–0.129) – (–0.028); MAR: β = –0.054, 95% CI: (–0.102) – (–0.014)]; Reflective System had a significant mediating effect between MAR and adolescent IA [β = –0.070, 95% CI: (–0.143) – (–0.023)]; Perceived Social Support and Impulsive System had a significant chain mediating effect between FAR/MAR and adolescent IA [FAR: β = –0.077, 95% CI: (–0.119) – (–0.050); MAR: β = –0.060, 95% CI: (–0.093) – (–0.039)]; the chain mediated effect of Perceived Social Support and Reflective System between FAR/MAR and adolescent IA effects were significant [FAR: β = –0.054, 95% CI: (–0.104) – (–0.024); MAR: β = –0.042, 95% CI: (–0.087) – (–0.017)]. In summary, several of this study’s hypotheses were validated.

**TABLE 4:** Total, direct, and indirect effects among the variables (*N* = 732).

Model paths	Standardized estimated value	*SE*	*p*	95% CI	
				Lower	Upper
**FAR**					
FAR→IA (total effect)	–0.271	0.035	<0.001	–0.342	–0.204
FAR→IA (direct effect)	—				
FAR→Perceived Social Support→IA	–0.065	0.032	0.038	–0.133	–0.004
FAR→Impulse System→IA	–0.075	0.026	0.002	–0.129	–0.028
FAR→Reflective System→IA	—				
FAR→Perceived Social Support→Impulse system→IA	–0.077	0.017	<0.001	–0.119	–0.050
FAR→Perceived Social Support→Reflective System→IA	–0.054	0.020	<0.001	–0.104	–0.024
**MAR**					
MAR→IA (total effect)	–0.276	0.037	<0.001	–0.347	–0.204
MAR→IA (direct effect)	—				
MAR→Perceived Social Support→IA	–0.050	0.025	0.035	–0.105	–0.004
MAR→Impulse System→IA	–0.054	0.023	0.008	–0.102	–0.014
MAR→Reflective System→IA	–0.070	0.031	<0.001	–0.143	–0.023
MAR→Perceived Social Support→Impulse system→IA	–0.060	0.014	<0.001	–0.093	–0.039
MAR→Perceived Social Support→Reflective System→IA	–0.042	0.017	<0.001	–0.087	–0.017

## Discussion

### Direct Relationship Between Father–Adolescent Relationships/Mother–Adolescent Relationships and Adolescent Internet Addiction

The purpose of this study was to examine the mechanism of the effect of FAR/MAR on adolescent IA and to compare the effect of FAR and MAR. In terms of the direct relationship between FAR/MAR and adolescent IA, both FAR and MAR demonstrated a significant negative correlation with IA, i.e., the stronger the FAR and MAR, the less likely adolescents were to develop IA, which was also consistent with prior research (Xu et al., 2014). However, inconsistent with existing research (Wang W. et al., 2018), the test results of the Hypothesis Model suggest that FAR and MAR do not have a direct effect on adolescent IA. One reason for this could be that this study did not directly analyze the parent-adolescent relationship as a separate variable when constructing the SEM for adolescent IA, but instead separated it into FAR and MAR to compare parent differences, weakening the direct effects of FAR and MAR on adolescent IA overall (Wang W. et al., 2018). Another reason could be that the FAR/MAR effect on adolescent IA in the multiple regression model is directed, whereas the effect of FAR/MAR on adolescent IA in the SEM contains indirect effects, and the coefficients of these mediating effects would somewhat attenuate the magnitude of the direct effects if they were larger (Iacobucci et al., 2007; Wang and Wang, 2019). Furthermore, Preacher and Hayes (2008) argued that the lack of a significant direct effect does not mean that FAR and MAR have no effect on IA, but rather that they influence adolescent IA primarily through mediating effects.

### Mediating Relations Between Father–Adolescent Relationships/Mother–Adolescent Relationships and Adolescent Internet Addiction

To elucidate the relationship between FAR/MAR and adolescent IA, a chain-mediated model was developed in this study. We discovered that Perceived Social Support and DSSC play a significant role in the association between FAR/MAR and adolescent IA. Reduced FAR/MAR specifically reduces adolescents’ Perceived Social Support, increasing their risk of IA. According to the deficiency-compensation theory, the primary reason adolescents become addicted to the Internet world is their inability to obtain emotional fulfillment in the real world. As a result, adolescents develop an increasing reliance on Internet socialization, Internet gaming, and Internet entertainment activities to compensate for unmet emotional needs in real life, which gradually results in IA (Gao and Chen, 2006). Thus, a lack of Perceived Social Support as a result of a decrease in FAR or MAR may be a significant factor in adolescent IA. Additionally, the lower FAR/MAR found in this study may increase the risk of IA by activating the Impulsive System and enhancing impulsivity in adolescents. Previous research has established a strong link between adolescence and impulsivity (Niv et al., 2012), and impulsivity in adolescence is primarily due to two physiological and environmental factors. With age, physiologically induced impulsivity decreases (Friedman et al., 2016; Pehlivanova et al., 2018). However, PBT suggests that problems in the living environment, particularly family problems, may play a significant role in adolescent impulsivity and ultimately lead to IA. A significant manifestation of family problems is the deterioration of the parent-adolescent relationship. Adolescents are prone to conflict with their parents, impairing their FAR or MAR (Chaplin et al., 2012). As a result of declining FAR or MAR, parental discipline and management of adolescents become more difficult, promoting impulsivity and ultimately increasing the risk of IA (Ding et al., 2017). In contrast, effective FAR and MAR can assist parents in communicating with their adolescents and reducing impulsivity, thereby suppressing adolescent IA (Liu et al., 2012; Martins et al., 2020).

More importantly, this study discovered that FAR/MAR enhanced adolescents’ Perceived Social Support, which in turn inhibited the Impulsive System’s activation and promoted the Reflective System’s activation, ultimately promoting adolescents’ self-control and lowering their risk of IA. According to the Dual-System Model of Self-Control, the impulsivity and Reflective Systems work in tandem to determine adolescents’ motivation for self-control. FAR/MAR can significantly improve adolescents’ perceptions of parental support (Karaer and Akdemir, 2019), thereby mitigating adolescents’ conflict with their parents and inhibiting impulsivity (Hamama and Ronen-Shenhav, 2012). Furthermore, increased parental support enables parents to carry out normal monitoring and parenting functions for their adolescents, reinforcing adolescents’ self-monitoring functions (Pilcher and Bryant, 2016). Thus, the processes contribute to the Reflective System’s dominance in the DSSC, which ultimately exhibits greater self-control (Zhang R. et al., 2021). In conclusion, this study demonstrates the importance of emphasizing both Perceived Social Support and DSSC when attempting to reduce the risk of IA in adolescents via FAR/MAR. Furthermore, if parents, teachers, or adolescents themselves wish to reduce the risk of adolescent IA via FAR/MAR, particular attention should be paid to the combined role of Perceived Social Support and DSSC.

### Differences Among Father–Adolescent Relationships, Mother–Adolescent Relationships to Adolescent Internet Addiction

The most significant and intriguing findings in this study suggest that the Reflective System mediates the relationship between MAR and adolescent IA, but that FAR does not affect adolescent IA via the Reflective System. i.e., MAR can enhance the activation of the adolescent Reflective System, thereby promoting self-monitoring and reflection and lowering the risk of IA in adolescents, whereas FAR does not. The current findings cast doubt on the hypothesis that the main method of preventing IA in adolescents is to enhance parent-adolescent relationship and that FAR acts similarly to MAR (Wang W. et al., 2018). In opposition to that, the current study suggests that MAR may play a more prominent role in promoting adolescent self-reflection and monitoring functions necessary for preventing IA. The presence of MAR is critical for the development of adolescent self-regulation, i.e., MAR facilitates adolescent self-monitoring, assessment, modification, and inhibition of their behavior or emotions more than parent-adolescent relationships or parenting styles do (Moilanen et al., 2010). Self-reflection and monitoring functions, on the other hand, enable adolescents to keep a watch on and monitor their Internet use, effectively lowering the risk of IA among adolescents (Leménager et al., 2016; Wang et al., 2021).

Additionally, we discovered that low FAR was associated with adolescent IA, but the association was less significant than the association with MAR, which is consistent with previous research (Xu et al., 2014). This finding may imply that MAR has a more pronounced and representative effect on adolescent IA than FAR. According to parental investment theory, fathers and mothers contribute differently to adolescent development. Mothers are primarily responsible for preventing adolescent emotional and behavioral development, whereas fathers are primarily responsible for ensuring the family’s proper functioning (Bjorklund and Kipp, 1996). Although FAR and MAR are essentially the same relationship and both are used to reflect the emotional cohesion between father/mother and adolescent (Russell and Saebel, 1997), parental involvement in parenting styles contributes to the FAR/differential MAR’s effects on adolescent IA. The differences in Perceived Social Support and DSSC elicited by FAR/MAR resulted in FAR/MAR having a different effect on adolescent IA. In diverse cultural contexts, a low FAR is more likely to trigger emotional and behavioral problems in adolescents than a high MAR (Baker, 2017; Pitsoane and Gasa, 2018). The primary reason for this may be that fathers exert less behavioral control over adolescents than mothers (Shek et al., 2018). Through their thoughts and emotions, mothers are more likely to convince adolescents that “IA is unacceptable behavior,” and good MAR facilitates this process of change. As a result, the presence of MAR is more important than the presence of FAR in promoting Reflective System activation and preventing IA in adolescents. When combined with the direct and indirect associations between FAR/MAR and adolescent IA found in this study, these findings suggest that FAR and MAR exert distinct effects on adolescent IA and that MAR is critical for reducing the risk of adolescent IA. From a cultural standpoint, the majority of Chinese adolescents regard fathers as serious and cognitive figures. Fathers frequently impose numerous behavioral restrictions on adolescents to teach them what they are not allowed to do, including restrictions on the use of Internet devices (Li and Lamb, 2013). This parental restraint contributes to adolescents’ reflection and monitoring of Internet device use, thereby lowering their risk of IA. However, when communication between fathers and adolescents breaks down for a variety of reasons, resulting in low FAR (Russell and Saebel, 1997; Pitsoane and Gasa, 2018), adolescents may become liberated from their fathers’ restraint, and FAR loses its ability to influence adolescents’ IA via the Reflective System.

Additional research is needed in the future to elucidate additional mechanisms underlying the effects of FAR/MAR on adolescent IA and to compare the effects of FAR and MAR. Mothers are more likely to influence adolescents’ perceptions of “IA as unacceptable behavior” through their thoughts and emotions, and a positive MAR aids in this process of change. As a result, the presence of MAR is more important than FAR in promoting Reflective System activation and preventing IA in adolescents. Taken together with the direct and indirect associations between FAR/MAR and adolescent IA found in this study, these findings suggest that FAR and MAR exert distinct effects on adolescent IA and that MAR is critical for reducing the risk of adolescent IA. Additional research is required in the future to elucidate additional mechanisms underlying the effects of FAR/MAR on adolescent IA and to compare the effects of FAR and MAR.

### Implications for Practical Services

To our knowledge, this is the first study to examine the mediating role of perceived and congregational support, as well as DSSC, in the relationship between FAR/MAR and adolescent IA, by comparing the effects of FAR and MAR on adolescent IA and contributing to our understanding of adolescent IA.

From a practical standpoint, our findings may contribute to the development of practical prevention and intervention strategies for reducing IA in adolescents. To begin, family intervention programs for adolescents with IA should be expanded to increase adolescents’ Perceived Social Support through FAR/MAR promotion. And, on this basis, adolescents should be guided toward developing self-reflective and monitoring abilities to rein in their impulsivity and thus reduce their risk of IA (Gunuc and Dogan, 2013). Second, and perhaps most importantly, different intervention plans should be developed for families with varying FAR/MAR statuses in various situations. For families with low FAR but a high MAR, priority can be given to fostering FAR to compensate for the perceived lack of social support and to promoting self-control to prevent adolescent IA (Liu et al., 2015). Priority should be given to fostering MAR in families with both low FAR and MAR, as MAR plays a critical role in inhibiting adolescent IA. Thirdly, the discovery that Perceived Social Support has a negative predictive effect on the Impulsive System while having a positive predictive effect on the Reflective System provides critical practice insights. To prevent and intervene with IA in adolescents, families, schools, and communities can establish a strong social support system (Shaheen et al., 2019). This system can help adolescents receive more social support, which can help reduce Impulsive System activation and promote the development of the Reflective System, which can help adolescents achieve self-control of IA.

### Limitations of This Study

This study is not without limitations. To begin, this study’s participants were concentrated in early adolescence. IA was more prevalent in early adolescence than in late adolescence (Moilanen et al., 2010). This means that the study’s findings may be slightly skewed and may not accurately reflect the general adolescent population. Second, this study focused primarily on the effect of perceived family support on adolescents’ IA. However, social support from school and community settings is critical in suppressing adolescent IA, and the family, school, and community all influence adolescent IA in different ways (Liu et al., 2015; Shaheen et al., 2019). As a result, additional research is required in the future to examine the effects of social support on adolescent IA in a variety of contexts. Third, only the cases of Perceived Social Support, Impulsivity System, and Reflective System as variable conditions were considered when comparing the differences in effects between FAR and MAR. MAR and FAR may also have opposite predictive effects on adolescent IA in other circumstances. As a result, the explanation for the causal relationship between FAR/MAR and adolescent IA becomes weaker. Future research should consider a longitudinal design that incorporates additional data on adolescent Internet use in order to reach more conclusive conclusions. Fourthly, future research could focus on family-centered approaches to adolescent IA interventions. Adolescents today face a complex and changing world filled with Internet device temptations, and not every adolescent is able to successfully avoid or overcome IA. Positive parental attitudes toward adolescents can contribute to the development of positive parent–adolescent relationships, which can significantly reduce or eliminate adolescent IA. Once adolescents develop an Internet addiction, parents should attempt to minimize the adolescent’s Internet identity. Parents’ love and trust motivate their children to overcome IA, and parents’ and children’s collaborative efforts are critical in assisting their children in overcoming IA.

## Data Availability Statement

The raw data supporting the conclusions of this article will be made available by the authors, without undue reservation.

## Ethics Statement

The studies involving human participants were reviewed and approved by Ethics Committee of Sichuan Normal University. Written informed consent to participate in this study was provided by the participants’ legal guardian/next of kin.

## Author Contributions

HQ: significant contribution to the design and writing of the study. QK: significant contribution to the writing and data collection and analysis. CB: significant contribution to the design and data collection aspects of the study. All authors: contributed to the article and approved the submitted version.

## Conflict of Interest

The authors declare that the research was conducted in the absence of any commercial or financial relationships that could be construed as a potential conflict of interest.

## Publisher’s Note

All claims expressed in this article are solely those of the authors and do not necessarily represent those of their affiliated organizations, or those of the publisher, the editors and the reviewers. Any product that may be evaluated in this article, or claim that may be made by its manufacturer, is not guaranteed or endorsed by the publisher.
